# Does omega-3 supplementation improve the inflammatory profile of patients with heart failure? a systematic review and meta-analysis

**DOI:** 10.1007/s10741-023-10327-0

**Published:** 2023-06-20

**Authors:** Konstantinos Prokopidis, Atiporn Therdyothin, Panagiotis Giannos, Jordi Morwani-Mangnani, Panagiotis Ferentinos, Alexandros Mitropoulos, Masoud Isanejad

**Affiliations:** 1 Department of Musculoskeletal Ageing and Science, Institute of Life Course and Medical Sciences, University of Liverpool, Liverpool, UK; 2 Society of Meta-Research and Biomedical Innovation, London, UK; 3 Department of Orthopedics, Police General Hospital, 492/1 Rama I Rd, Pathum Wan, Pathum Wan District, Bangkok, Thailand; 4 Department of Life Sciences, Faculty of Natural Sciences, Imperial College London, London, UK; 5 Section of Molecular Epidemiology, Department of Biomedical Data Sciences, Leiden University Medical Center, Leiden, Netherlands; 6 Carnegie School of Sport, Leeds Beckett University, Leeds, UK; 7 Lifestyle, Exercise and Nutrition Improvement (LENI) Research Group, Department of Nursing and Midwifery, Sheffield Hallam University, Sheffield, UK

**Keywords:** Omega-3, Heart failure, Inflammation, TNF-a, IL-6, CRP

## Abstract

Omega-3 fatty acids are potential anti-inflammatory agents that may exert beneficial outcomes in diseases characterised by increased inflammatory profile. The purpose of this study was to comprehensively evaluate the existing research on the effectiveness of n-3 fatty acid supplementation in lowering levels of circulating inflammatory cytokines in patients with heart failure (HF). From the beginning until October 2022, randomised controlled trials (RCTs) were the subject of PubMed, Scopus, Web of Science, and Cochrane Library literature search. Omega-3 fatty acid supplementation vs. placebo were compared in eligible RCTs to see how they affected patients with HF in terms of inflammation, primarily of tumour necrosis factor-alpha (TNF-a), interleukin-6 (IL-6), and c-reactive protein (CRP). A meta-analysis employing the random effects inverse-variance model and standardised mean differences was performed to assess group differences. Ten studies were included in this systematic review and meta-analysis. Our main analysis (*k* = 5) revealed a beneficial response of n-3 fatty acid supplementation on serum TNF-a (SMD: − 1.13, 95% CI: − 1.75– − 0.50, *I*^2^ = 81%, *P* = 0.0004) and IL-6 levels (*k* = 4; SMD: − 1.27, 95% CI: − 1.88– − 0.66, *I*^2^ = 81%, *P* < 0.0001) compared to placebo; however, no changes were observed in relation to CRP (*k* = 6; SMD: − 0.14, 95% CI: − 0.35–0.07, *I*^2^ = 0%, P = 0.20). Omega-3 fatty acid supplementation may be a useful strategy for reducing inflammation in patients with HF, but given the paucity of current studies, future studies may increase the reliability of these findings.

**Supplementary Information:**

The online version contains supplementary material available at 10.1007/s10741-023-10327-0.

## Introduction

Heart failure (HF) is a clinical illness brought on by anatomical and functional myocardial abnormalities that limit ventricular filling or blood ejection [1, 2]. While hemodynamic and neurohormonal responses are triggered to ensure enough tissue perfusion in order to counteract this ventricular dysfunction, their continued activation over the long term results in structural and functional damage [3]. HF is divided in three distinct phenotypes based on left ventricular ejection fraction (LVEF), particularly, reduced (HFrEF; ≤ 40% EF), preserved (HFpEF; ≥ 50% EF), and mid-range (HFmrEF; 41–49% EF) [1, 2]. Differentiation of patients with HF based on LVEF is important due to different underlying aetiologies, demographics, co-morbidities, and response to therapies [4].

In HFrEF, myocardial injury activates the inflammatory responses regulated by the innate and adaptive immune system [5, 6]. The inflammatory responses are described by an initial increase in proinflammatory cytokines and chemokines in conjunction with infiltration of neutrophils and monocytes into the damaged myocardium [6]. Inflammation is considered a critical pathophysiological factor in HF, predicting poor prognosis independent of LVEF [7]. Some notable inflammatory biomarkers in HF include tumour necrosis factor alpha (TNF-a), interleukin 1 (IL-1), interleukin-6 (IL-6), interleukin-8 (IL-8), myeloperoxidase (MPO), inducible nitric oxide synthase (iNOS), and c-reactive protein (CRP) [3]. Several interventional trials have attempted to target or regulate the activity of those inflammatory markers. Indeed, medical treatment has shown promising results to reduce inflammation in people with HF [7], while following a healthy diet has also displayed a beneficial impact [1]. However, whether non-pharmacological interventions such as dietary supplements could benefit patients with HF is currently underexplored.

Attention to the role of n-3 fatty acids in human health and disease has been continuously increased during recent decades. Many clinical and epidemiological studies have shown a positive role for n-3 fatty acids in cardiovascular disease outcomes [8]. More specifically, n-3 fatty acids such as eicosapentaenoic acid (EPA) and docosahexaenoic acid (DHA) have been reported to modulate the expression of genes involved in the nuclear factor-kappa B (NF-κB) pathway [9]. A recent systematic review and meta-analysis assessed the effect of n-3 fatty acids on left ventricular remodelling in HF [10], indicating that a n-3 dosage of ≥ 600 g/day could decrease serum TNF-a, IL-6, and high sensitivity-CRP (hsCRP) [10]. Nevertheless, the robustness of this systematic review and meta-analysis is questioned due to inflammatory outcomes being secondary outcomes of interest; hence, there was (i) inclusion of non-placebo treated and/or non-randomised controlled studies, (ii) the lack of subgroup analysis based on duration, dose, age, and type of HF, and (iii) the lack of sensitivity analyses.

The aim of this study was to systematically assess the published literature pertinent to the efficacy of n-3 fatty acid supplementation in reducing the inflammatory cytokine levels of TNF-a, IL-6, and CRP, in patients with HF. A secondary aim was to assess the effect of n-3 fatty acid supplementation on IL-1, intercellular adhesion molecule 1 (ICAM-1), vascular adhesion molecule 1 (VCAM-1), and monocyte chemoattractant protein-1 (MCP-1), which also play a central role in the regulation of inflammatory responses.

## Methods

This systematic review and meta-analysis were conducted in accordance with the PRISMA (Preferred Reporting Items for Systematic Reviews and Meta-Analyses) guidelines [11]. The protocol was registered in the PROSPERO (International Prospective Register of Systematic Reviews) database (CRD:42,022,367,713).

### Search strategy

Two independent reviewers (KP and PG) searched PubMed, Scopus, Web of Science, and the Cochrane library from inception until October 2022. A search strategy involving the following terms was used: “heart failure” OR “ejection fraction” AND “omega-3*” OR “n-3.” A manual search of references cited in the selected articles and published reviews was also performed. Discrepancies in the literature search process were resolved by a third investigator (MI). Studies were included according to the PICOS (Population, Intervention, Comparator, Outcomes, and Study design) process (Table S1). The following inclusion criteria were applied: (1) studies were randomised controlled trials (RCTs); (2) population comprised of patients with HF; (3) intervention group received n-3 supplementation; and (4) control group received a placebo. Studies were excluded if (1) they were not RCTs; and (2) a full text was not available.

### Data extraction and risk of bias

Two authors (KP and JM) extracted data independently. Extracted data included the name of first author, country, date of publication, study design, age, sex, type of HF, number of participants, outcomes measured, dose and duration of treatment, and whether dietary intake and serum omega-3 levels were assessed. Disagreements between authors were resolved by a third reviewer (PG). The quality of the included studies was assessed using version 2 of the Cochrane risk-of-bias 2 tool for randomised trials (RoB 2) and evaluated by two independent reviewers (PF and AM). Appraisal of risk of bias using the RoB 2 tool included the assessment of the following domains of bias in RCTs: (1) randomisation process, (2) deviations from intended interventions, (3) missing outcome data, (4) measurement of the outcome, and (5) selection of the reported result. In accordance with the RoB 2 tool scoring system, study quality was defined as low risk of bias, some concerns, or high risk of bias.

### Statistical analysis

In this meta-analysis, quantitative data were treated as continuous measurements, and changes in outcomes from baseline to follow-up were compared between groups to calculate mean differences. When units of measurements were inconsistent and could not be converted to units required to be included in the analysis, standardised mean differences were used. Added to this, we combined studies that used both normal and similarly high-sensitivity markers of inflammation (i.e., trials that assessed hsCRP with CRP), considering their identical outcomes. Statistical significance was assessed using the random-effects model and the inverse-variance method. Any changes between baseline and follow-up outcome measurements for which standard deviations were missing were estimated by calculating a correlation coefficient from a known change from the baseline standard deviation derived from a similar study.

Statistical heterogeneity of outcome measurements between different studies was assessed using the overlap of their 95% confidence intervals (95%CIs) and expressed as a measurement of Cochran’s *Q* (*χ*^2^ test) and *I*^2^. Data were classified as moderately heterogeneous when *I*^2^ values ranged from 50 to 74.9% and as highly heterogeneous when values were 75% and above. Furthermore, sensitivity analysis was performed to evaluate the robustness of reported statistical results by discounting the effects of the HFmrEF and the effects of olive oil as placebo. Subgroup analyses based on age (< 65 years vs. ≥ 65 years), treatment duration (≤ 12 weeks vs. > 12 weeks), dose (low dose < 2 g/day vs. high dose ≥ 2 g/day), and type of HF (acute vs. chronic) were also performed. The meta-analysis of data was synthesised using Cochrane’s Review Manager (RevMan 5.4.1) software.

## Results

### Search results

**Fig. 1:**
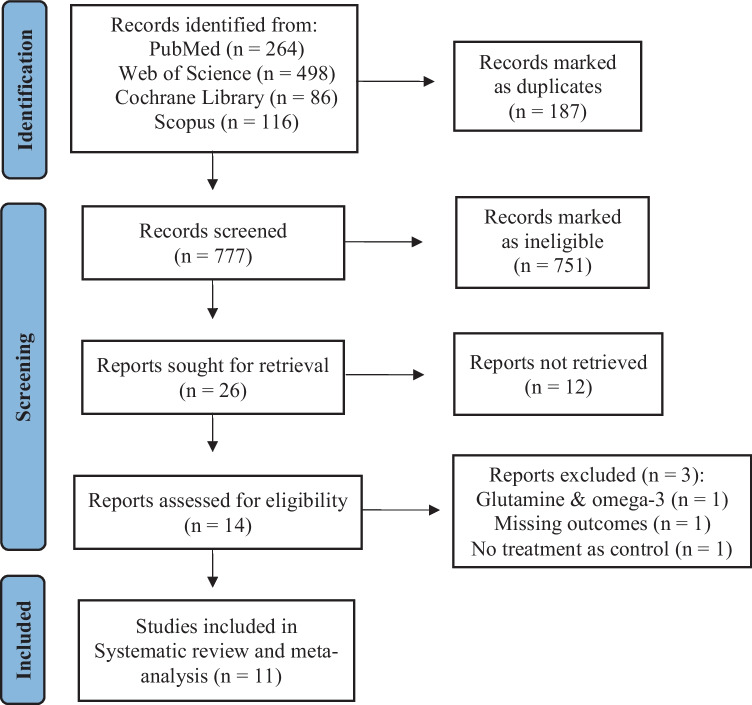
Flowchart of the literature search

Our initial literature search tallied 964 publications. After the exclusion of 187 duplicates, 777 unique publications were screened. Overall, 751 publications were marked as ineligible, while full-text screening of the remaining 26 publications resulted in 14 eligible RCTs examining n-3 fatty acid supplementation on patients with HF. Finally, 11 RCTs were included in the systematic review and meta-analysis [12-22], for which one could not be included in our meta-analysis due to inadequate data [22]. Regarding the excluded studies, one study was missing baseline values, one study used coadministration of n-3 with glutamine, and one study used no treatment as control (Fig. 1).

### Characteristics of the included studies

A comprehensive description of the included studies is presented in Table 1.

**Table 1:** Study and participant characteristics of the included studies in the meta-analysis

**Study year**	**Country**	**Study design**	**Total**		**Omega-3**		**Placebo**		**Treatment dose (g/day)**	**Treatment duration**	**Control group**	**Outcomes**
			***n*** **(M/F)**	**Age**	***n*** **(M/F)**	**Age**	***n*** **(M/F)**	**Age**				
Palomas 2021	Spain	Double-blind RCT	43 (31/12)	75.5 (8.7)	20 (14/6)	76 (7.7)	23 (17/6)	75 (9.6)	4	4 weeks	Placebo (gelatin)	CRP
Oikonomou 2018	Greece	Double-blind cross-over trial	31 (23/8)	66.5 (5.5)	15 (11/4)	66 (4)	16 (12/4)	67 (7)	2	8 weeks (× 2) + 6-week washout period	Placebo (olive oil)	hsCRP
Wurm 2018	Austria	Double-blind RCT	40 (34/6)	59.7 (8.0)	1 g: 12 (10/2) 4 g: 12 (12/0)	1 g: 59 (54–63) 4 g: 64 (58–66)	16 (12/4)	56 (46–63)	1 or 4	12 weeks	Placebo	CRP
Makarewicz-Wujec 2017	Poland	Double-blind RCT	30 (26/4)	64.56 (1.06)	15 (13/2)	67.5 (0.7)	15 (13/2)	62.06 (1.41)	1	12 weeks	Placebo (corn oil)	hsCRP MCP-1
Heydari 2016	USA	Double-blind RCT	358 (288/70)		180 (148/32)	60 (10)	178 (140/38)	58 (10)	4	6 months	Placebo (corn oil)	hsCRP
Moertl 2011	Austria	Double-blind RCT	43 (37/6)	58.7 (9.8)	1 g: 14 (12/2), 4 g: 13 (13/0)	1 g: 59 (7), 4 g: 61.9 (9.6)	16 (12/4)	55.1 (12.7)	1 or 4	12 weeks	Placebo (gelatin)	TNF-a IL-6
Nodari 2011	USA	Double-blind RCT	133 (120/13)	62.5 (10.0)	67 (64/3)	61 (11)	66 (56/10)	64 (9)	2	12 months	Placebo (olive oil)	TNF-a IL-6 IL-1
Eschen 2010	Italy	Double-blind RCT	138 (118/20)	59.5 (9.0)	69 (57/12)	58 (10)	69 (61/8)	61 (8)	0.9	24 weeks	Placebo (olive oil)	hsCRP ICAM-1 VCAM-1
Nodari 2009	Italy	Double-blind RCT	44 (40/4)	62.96 (10.29)	22 (21/1)	61.09 (11.22)	22 (19/3)	64.82 (9.46)	1	6 months	Placebo (olive oil)	TNF-a IL-6 IL-1
Zhao 2009	China	Single-blind RCT	75 (55/20)	72.5 (8.0)	38 (27/11)	74 (6)	37 (28/9)	71 (10)	2	3 months	Placebo	TNF-a IL-6 hsCRP ICAM-1 VCAM-1
Mehra 2006	USA	Double-blind RCT	14 (12/2)	60.5 (7.5)	7 (6/1)	59 (7)	7 (6/1)	61 (8)	8	18 weeks	Placebo (corn oil)	TNF-a IL-1

*CRP* c-reactive protein, *hsCRP* high-sensitivity c-reactive protein, *ICAM-1* intercellular adhesion molecule 1, *IL* interleukin, *MCP-1* monocyte chemoattractant protein-1, *TNF-a* tumour necrosis factor-alpha, *VCAM-1* vascular cell adhesion molecule 1

Values are expressed as mean ± SD

### Risk of bias assessment

Of the included studies, four studies were classified as having overall some concerns [12, 16, 20, 21], due to unclear description of randomisation process, and as to whether participants were aware of the intervention. In one study [21], the intervention group was comprised of patients with greater body mass index compared to controls. In addition, in the same study, there was no clear description on blinding of the investigators [21]. The rest of the studies were identified having low risk [13–15, 17–19, 22]. A detailed traffic light plot is presented in Table S2.

### Omega-3 supplementation and TNF-a

**Fig. 2:**
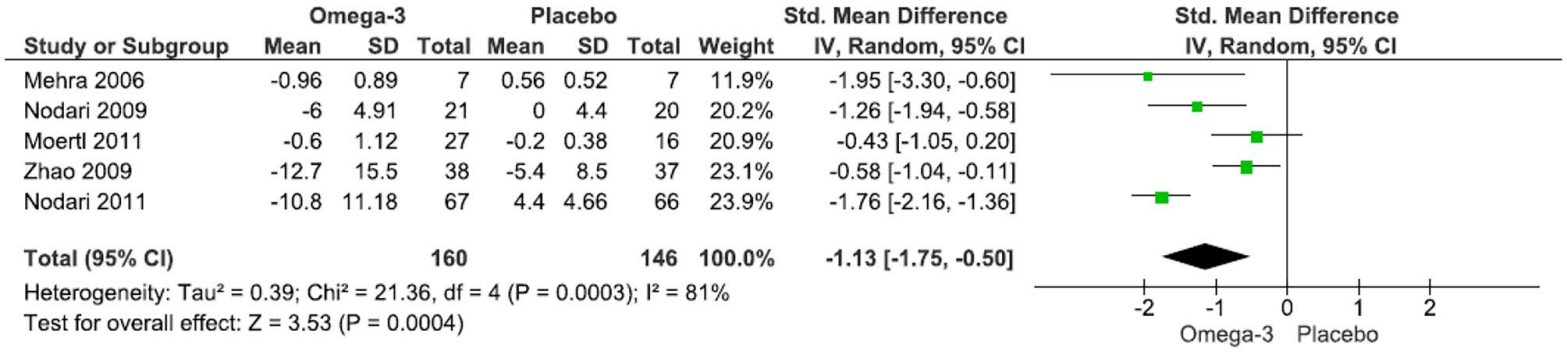
Effect of n-3 fatty acid supplementation on TNF-a levels in patients with HF

Our main analysis (*k* = 5) revealed a significant impact of n-3 fatty acid supplementation to lower TNF-a levels compared to placebo, showing a high degree of heterogeneity (SMD: − 1.13, 95% CI: − 1.75– − 0.50, *I*^2^ = 81%, *P* = 0.0004) (Fig. 2). Subgroup analysis showed a significant change by lower dose (*k* = 3; SMD: − 1.20, 95% CI: − 1.98– − 0.43, *I*^2^ = 86%, *P* = 0.002), but not using higher doses (*k* = 2; SMD: − 1.07, 95% CI: − 2.54–0.41, *I*^2^ = 75%, *P* = 0.16) (Fig. S1). In addition, longer treatment duration (*k* = 4; SMD: − 1.31, 95% CI: − 2.00– − 0.62, *I*^2^ = 80%, *P* = 0.0002) (Fig. S2) and being below 65 years of age (*k* = 4; SMD: − 1.29, 95% CI: − 2.00– − 0.59, *I*^2^ = 77%, *P* = 0.0003) (Fig. S3) were significant contributors in lowering TNF-a levels compared to placebo.

### Omega-3 supplementation and IL-6

**Fig. 3:**
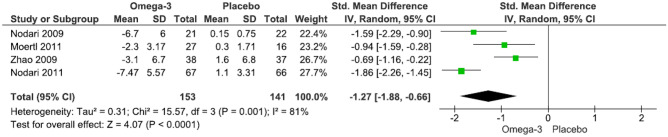
Effect of n-3 fatty acid supplementation on IL-6 levels in patients with HF

Our main analysis (*k* = 4) revealed a significant impact of n-3 supplementation on suppressing IL-6 levels compared to placebo with a high degree of heterogeneity (SMD: − 1.27, 95% CI: − 1.88– − 0.66, *I*^2^ = 81%, *P* < 0.0001) (Fig. 3). Subgroup analysis showed a significant change by lower dose (*k* = 3; SMD: − 1.37, 95% CI: − 2.15– − 0.60, *I*^2^ = 86%, *P* = 0.0005) (Fig. S4), longer treatment duration (*k* = 3; SMD: − 1.37, 95% CI: − 2.15– − 0.60, *I*^2^ = 86%, *P* = 0.0005) (Fig. S5), and patients below 65 years of age (*k* = 3; SMD: − 1.50, 95% CI: − 2.05– − 0.95, *I*^2^ = 64%, *P* < 0.0001) (Fig. S6).

### Omega-3 supplementation and CRP

**Fig. 4:**
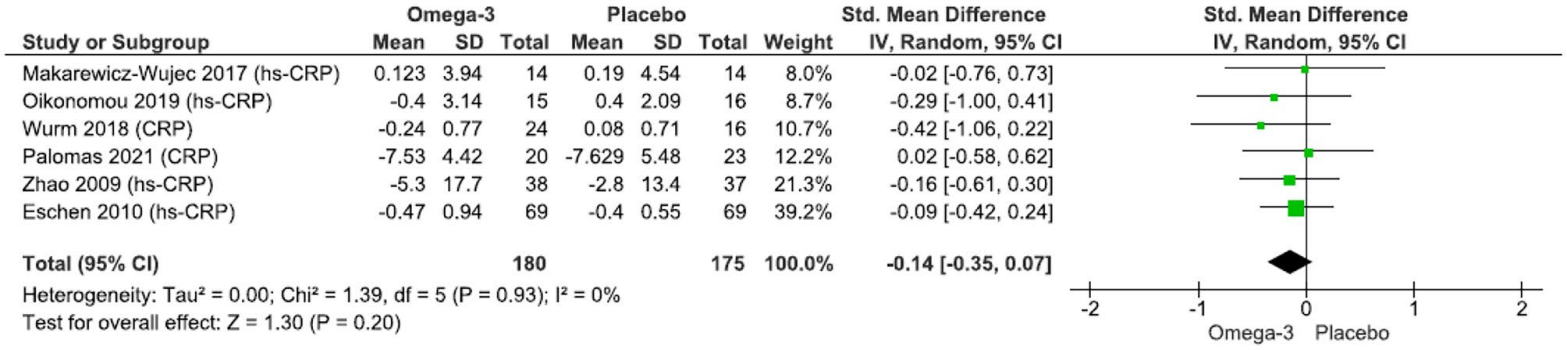
Effect of n-3 fatty acid supplementation on CRP levels in patients with HF

Our main analysis (*k* = 6) showed no significant differences between n-3 supplementation and placebo on CRP, analysing studies with low degree of heterogeneity (SMD: − 0.14, 95% CI: − 0.35–0.07, *I*^2^ = 0%, *P* = 0.20) (Fig. 4). Subgroup analysis showed no significant changes by lower (*k* = 4; SMD: − 0.12, 95% CI: − 0.36–0.11, *I*^2^ = 0%, *P* = 0.31) or higher dose (*k* = 2; SMD: − 0.19, 95% CI: − 0.62–0.25, *I*^2^ = 0%, *P* = 0.40) (Fig. S7), type of acute (*k* = 3; SMD: − 0.14, 95% CI: − 0.52–0.23, *I*^2^ = 0%, *P* = 0.46) or chronic HF (*k* = 3; SMD: − 0.14, 95% CI: − 0.39–0.11, *I*^2^ = 0%, *P* = 0.29) (Fig. S8), and patients 65 years of age and above (*k* = 5; SMD: − 0.17, 95% CI: − 0.44–0.10, *I*^2^ = 0%, *P* = 0.22) (Fig. S9). Sensitivity analysis based on LVEF > 40% (one study had a mean of 42%) and a concerning risk for bias [21] did not alter the findings of the main analysis (*k* = 5; SMD: − 0.15, 95% CI: − 0.37–0.07, *I*^2^ = 0%, *P* = 0.18) (Fig. S10). Likewise, exclusion of two studies due to having olive oil as placebo [15, 20] showed no changes (*k* = 4; SMD: − 0.15, 95% CI: − 0.44–0.14, *I*^2^ = 0%, *P* = 0.32) (Fig. S11).

Due to inadequate data for which one study provided only the differences between the treatment vs. placebo [22], levels of CRP followed a decreasing trend following a 4 g daily dose of omega-3 supplementation (− 25% vs. placebo), albeit no statistically significant differences occurred.

### Omega-3 supplementation and IL-1, ICAM-1, and vICAM-1

Our main analysis (*k* = 3) showed no significant differences between n-3 supplementation and placebo on IL-1, using RCTs of high degree of heterogeneity (SMD: − 0.94, 95% CI: − 2.66–0.78, *I*^2^ = 95%, *P* = 0.28) (Fig. S12). Additionally, similar results were found regarding ICAM-1 and vICAM-1 (*k* = 2) (SMD: − 0.19, 95% CI: − 0.95–0.56, *I*^2^ = 86%, *P* = 0.61) (Fig. S13). Subgroup analysis based on lower n-3 dose showed a significant reduction of IL-1 compared to placebo, although a high degree of heterogeneity was identified (*k* = 2; SMD: − 1.81, 95% CI: − 3.23– − 0.40, *I*^2^ = 92%, *P* = 0.01) (Fig. S14).

## Discussion

In this study, we reviewed RCTs investigating the effect of n-3 fatty acid supplementation on alleviating inflammatory cytokine levels in patients with HF. Our main analysis revealed that n-3 fatty acid supplementation suppresses circulating TNF-a and IL-6 levels. Subgroup analyses identified a beneficial effect of supplementation in patients aged < 65 years, longer treatment duration (> 12 weeks), and lower dose (< 2 g/day), in both cytokines. There was no significant effect of n-3 fatty acid supplementation on serum CRP, IL-1, ICAM-1, and VCAM-1.

### Findings from subgroup analyses

Our subgroup analysis based on the dose of n-3 fatty acid supplementation showed that lower dose (< 2 g/day) led to a significant suppression of TNF-a and IL-6, while a higher dose (≥ 2 g/day) did not. Although a biological rationale behind this finding has not been explored, we speculate that the high 95% CIs and SD values of the low number (*k* = 2) of studies and sample size included in this subgroup analysis could explain these contrasting results. In the study by Moertl et al. (2011), the authors utilised a lower dose of n-3 supplementation (1 g/day) in 14 participants, showing statistically similar findings (*P* = 0.17), with a minor clinical difference compared to the higher dose of 4 g/day (*P* = 0.07) (1 g/day—pre: 2.6 (2.4–3.29) to post: 2.4 (1.9–2.5) pg/mL; 4 g/day—pre: 2.1 (1.6–2.6) to post: 1.5 (0.1–2.1) pg/mL; *n* = 13) [19]. It may be more illuminating to determine whether a larger number of studies demonstrating that high n-3 doses have a negligible impact on TNF-a could yield a statistically significant result in a future meta-analysis or whether a biological mechanism of increased doses could negate its efficacy in lowering this inflammatory marker.

From previous studies, mechanistically, n-3 fatty acids displace arachidonic acid on cell membranes and produce eicosanoids that are deemed anti-inflammatory [23]. N-3 fatty acids also downregulate the expression of cyclooxygenase (COX); a key enzyme in production of pro-inflammatory cytokines by inhibition of NF-kB signalling [24]. Previous research has also showed that n-3 fatty acid supplementation has resulted in lower levels of inflammatory cell infiltration and fibrosis in cardiac tissue in murine models [25] and has augmented the polarisation of the anti-inflammatory M2 macrophage, resulting in cardiac remodelling [25, 26].

Moreover, while our subgroup analysis demonstrated a significant reduction in TNF-a and IL-6 in trials with longer treatment duration (> 12 weeks), only one study included in our main analysis followed a shorter term duration (≤ 12 weeks) [19]. In particular, this study revealed a significant reduction of IL-6 with 4 g/day of n-3 fatty acid supplementation, although a non-significant but decreasing trend with 1 g/day. Albeit these findings may support a higher dose to reduce IL-6 levels, the same study found a non-significant decrease in TNF-a utilising a higher dose (4 g/day). Therefore, in spite of the low sample size in this study, it is worth stating that the non-statistically significant result in the subgroup analysis utilising higher doses of n-3 fatty acids may had also been impacted by insufficient treatment duration.

Our subgroup analysis based on age showed a reduction of serum TNF-a and IL-6 in patients with HF aged < 65 years. However, there was only one RCT demonstrating a significant decrease in serum TNF-a and IL-6 in patients ≥ 65 years following 3 months of 2 g/day n-3 fatty acid supplementation [13], and as such, these results cannot be extrapolated in patients aged ≥ 65 years. Interestingly, longitudinal research in patients with HF above 65 years has demonstrated that ageing is a significant contributor to higher levels of inflammatory markers and is linked to a greater risk of HF development [27]. Therefore, further clinical trials are required to explore whether patients aged ≥ 65 years would experience similar reductions in TNF-a and IL-6.

### Strengths and limitations

To our knowledge, this is the first meta-analysis of RCTs with the main aim to investigate the effect of n-3 fatty acid supplementation on pro-inflammatory cytokines in patients with HF. HF in patients aged < 65 years may have a different aetiology compared to their older counterparts [28] and, hence, different response to the anti-inflammatory effects of n-3 fatty acids. Therefore, our results may be represented more accurately in patients with HF aged < 65 years, given that middle-aged (i.e., < 65 years) and older (i.e., ≥ 65 years) patients with HF may display similar cardiac remodelling during disease progression but differing inflammatory profiles [29]. In addition, our analysis was limited by a small number of available RCTs to draw conclusions pertinent to lower vs. higher dose and shorter vs. longer treatment duration. Hence, the results from subgroup analyses should be interpreted with caution due to the limited number of studies (i.e., *k* = 3 for lower dose, and *k* = 2 for higher dose; regarding TNF-a).

## Conclusion

This systematic review and meta-analysis demonstrated the potential of n-3 fatty acid supplementation to suppress markers of inflammation in patients with HF. Whether these results may be applicable to only lower doses, longer treatment duration, and patients aged < 65 years have yet to be investigated, concerning the limited research around this field. Supplementation with n-3 fatty acids is an inexpensive non-pharmacological agent; hence, more research on the topic could yield prominent findings pertaining to HF treatment by mediating patients’ inflammatory response.

### Supplementary Information

Below is the link to the electronic supplementary material.

Supplementary file1 (DOCX 14 KB)

Supplementary file2 (DOCX 566 KB)

Supplementary file3 (DOCX 75 KB)

Supplementary file4 (DOCX 74 KB)

Supplementary file5 (DOCX 74 KB)

Supplementary file6 (DOCX 70 KB)

Supplementary file7 (DOCX 71 KB)

Supplementary file8 (DOCX 70 KB)

Supplementary file9 (DOCX 73 KB)

Supplementary file10 (DOCX 73 KB)

Supplementary file11 (DOCX 72 KB)

Supplementary file12 (DOCX 55 KB)

Supplementary file13 (DOCX 52 KB)

Supplementary file14 (DOCX 43 KB)

Supplementary file15 (DOCX 38 KB)

Supplementary file16 (DOCX 64 KB)

## Data Availability

The authors confirm that the data supporting the findings of this study are available within the article and/or its supplementary materials.

## References

[1] McDonagh TA, Metra M, Adamo M, Gardner RS, Baumbach A, Böhm M, Burri H, Butler J, Čelutkienė J, Chioncel O, Cleland JGF, Coats AJS, Crespo-Leiro MG, Farmakis D, Gilard M, Heymans S, Hoes AW, Jaarsma T, Jankowska EA, Lainscak M, Lam CSP, Lyon AR, McMurray JJV, Mebazaa A, Mindham R, Muneretto C, Francesco Piepoli M, Price S, Rosano GMC, Ruschitzka F, Kathrine Skibelund A (2021) ESC Guidelines for the diagnosis and treatment of acute and chronic heart failure. Eur Heart J 42(36):3599–3726

[2] Ponikowski P, Voors AA, Anker SD, Bueno H, Cleland JG, Coats AJ, Falk V, González-Juanatey JR, Harjola V-P, Jankowska EA (2016). 2016 ESC Guidelines for the diagnosis and treatment of acute and chronic heart failure. Kardiologia Polska (Polish Heart Journal).

[3] Reina-Couto M, Pereira-Terra P, Quelhas-Santos J, Silva-Pereira C, Albino-Teixeira A, Sousa T (2021). Inflammation in human heart failure: major mediators and therapeutic targets. Front Physiol.

[4] Butler J, Fonarow GC, Zile MR, Lam CS, Roessig L, Schelbert EB, Shah SJ, Ahmed A, Bonow RO, Cleland JG (2014) Developing therapies for heart failure with preserved ejection fraction: current state and future directions. JACC: Heart Failure 2(2):97–112 10.1016/j.jchf.2013.10.006 PMC4028447 24720916

[5] Van Linthout S, Tschöpe C (2017). Inflammation - cause or consequence of heart failure or both?. Curr Heart Fail Rep.

[6] Adamo L, Rocha-Resende C, Prabhu SD, Mann DL (2020). Reappraising the role of inflammation in heart failure. Nat Rev Cardiol.

[7] Murphy SP, Kakkar R, McCarthy CP, Januzzi JL Jr (2020) Inflammation in heart failure: JACC state-of-the-art review. J Am Coll Cardiol 75(11):1324–1340 10.1016/j.jacc.2020.01.014 32192660

[8] Khan SU, Lone AN, Khan MS, Virani SS, Blumenthal RS, Nasir K, Miller M, Michos ED, Ballantyne CM, Boden WE, Bhatt DL (2021). Effect of omega-3 fatty acids on cardiovascular outcomes: a systematic review and meta-analysis. EClinicalMedicine.

[9] Allam-Ndoul B, Guénard F, Barbier O, Vohl MC (2016) Effect of n-3 fatty acids on the expression of inflammatory genes in THP-1 macrophages. Lipids Health Dis 15:69 10.1186/s12944-016-0241-4 PMC4820929 27044314

[10] Liu J, Meng Q, Zheng L, Yu P, Hu H, Zhuang R, Ge X, Liu Z, Liang X, Zhou X (2022) Effect of omega-3 polyunsaturated fatty acids on left ventricular remodeling in chronic heart failure: a systematic review and meta-analysis. Br J Nutr 1–35 10.1017/S0007114521004979 35241186

[11] Page MJ, McKenzie JE, Bossuyt PM, Boutron I, Hoffmann TC, Mulrow CD, Shamseer L, Tetzlaff JM, Akl EA, Brennan SE, Chou R, Glanville J, Grimshaw JM, Hróbjartsson A, Lalu MM, Li T, Loder EW, Mayo-Wilson E, McDonald S, McGuinness LA, Stewart LA, Thomas J, Tricco AC, Welch VA, Whiting P, Moher D (2021) The PRISMA 2020 statement: an updated guideline for reporting systematic reviews. Bmj 372:n71 10.1136/bmj.n71 PMC8005924 33782057

[12] Mehra MR, Lavie CJ, Ventura HO, Milani RV (2006). Fish oils produce anti-inflammatory effects and improve body weight in severe heart failure. J Heart Lung Transplant.

[13] Zhao YT, Shao L, Teng LL, Hu B, Luo Y, Yu X, Zhang DF, Zhang H (2009) Effects of n-3 polyunsaturated fatty acid therapy on plasma inflammatory markers and N-terminal pro-brain natriuretic peptide in elderly patients with chronic heart failure. J Int Med Res 37(6):1831–1841 10.1177/147323000903700619 20146881

[14] Bonilla Palomas JL, Gámez López AL, Moreno Conde M, López Ibáñez MC, Moreno Villar MA (2021) [Effect of omega-3 fatty acids on hypoalbuminemia in acute heart failure patients with increased inflammatory activity]. Nutr Hosp 38(5):890–896 10.20960/nh.03637 34154367

[15] Oikonomou E, Vogiatzi G, Karlis D, Siasos G, Chrysohoou C, Zografos T, Lazaros G, Tsalamandris S, Mourouzis K, Georgiopoulos G, Toutouza M, Tousoulis D (2019). Effects of omega-3 polyunsaturated fatty acids on fibrosis, endothelial function and myocardial performance, in ischemic heart failure patients. Clin Nutr.

[16] Nodari S, Triggiani M, Campia U, Manerba A, Milesi G, Cesana BM, Gheorghiade M, Dei Cas L (2011) Effects of n-3 polyunsaturated fatty acids on left ventricular function and functional capacity in patients with dilated cardiomyopathy. J Am Coll Cardiol 57(7):870–879 10.1016/j.jacc.2010.11.017 21215550

[17] Nodari S, Metra M, Milesi G, Manerba A, Cesana BM, Gheorghiade M, Dei Cas L (2009) The role of n-3 PUFAs in preventing the arrhythmic risk in patients with idiopathic dilated cardiomyopathy. Cardiovas Drugs Ther 23(1):5–15 10.1007/s10557-008-6142-7 18982439

[18] Wurm R, Schrutka L, Hammer A, Moertl D, Berger R, Pavo N, Lang IM, Goliasch G, Huelsmann M, Distelmaier K (2018). Polyunsaturated fatty acids supplementation impairs anti-oxidant high-density lipoprotein function in heart failure. Eur J Clin Invest.

[19] Moertl D, Hammer A, Steiner S, Hutuleac R, Vonbank K, Berger R (2011). Dose-dependent effects of omega-3-polyunsaturated fatty acids on systolic left ventricular function, endothelial function, and markers of inflammation in chronic heart failure of nonischemic origin: a double-blind, placebo-controlled, 3-arm study. Am Heart J.

[20] Eschen O, Christensen JH, Romano P, Sala P, Schmidt EB (2010) Effects of marine n-3 fatty acids on circulating levels of soluble adhesion molecules in patients with chronic heart failure. Cell Mol Biol (Noisy-le-grand) 56(1):45–51 20196969

[21] Makarewicz-Wujec M, Parol G, Parzonko A, Kozłowska-Wojciechowska M (2017). Supplementation with omega-3 acids after myocardial infarction and modification of inflammatory markers in light of the patients’ diet: a preliminary study. Kardiol Pol.

[22] Heydari B, Abdullah S, Pottala JV, Shah R, Abbasi S, Mandry D, Francis SA, Lumish H, Ghoshhajra BB, Hoffmann U (2016). Effect of omega-3 acid ethyl esters on left ventricular remodeling after acute myocardial infarction: the OMEGA-REMODEL randomized clinical trial. Circulation.

[23] Simonetto M, Infante M, Sacco RL, Rundek T, Della-Morte D (2019) A novel anti-inflammatory role of omega-3 PUFAs in prevention and treatment of atherosclerosis and vascular cognitive impairment and dementia. Nutrients 11:10 10.3390/nu11102279 PMC6835717 31547601

[24] Massaro M, Habib A, Lubrano L, Del Turco S, Lazzerini G, Bourcier T, Weksler BB, De Caterina R (2006) The omega-3 fatty acid docosahexaenoate attenuates endothelial cyclooxygenase-2 induction through both NADP(H) oxidase and PKC epsilon inhibition. Proc Natl Acad Sci U S A 103(41):15184–15189 10.1073/pnas.0510086103 PMC1622797 17018645

[25] Toko H, Morita H, Katakura M, Hashimoto M, Ko T, Bujo S, Adachi Y, Ueda K, Murakami H, Ishizuka M, Guo J, Zhao C, Fujiwara T, Hara H, Takeda N, Takimoto E, Shido O, Harada M, Komuro I (2020) Omega-3 fatty acid prevents the development of heart failure by changing fatty acid composition in the heart. Sci Rep 10(1):15553 10.1038/s41598-020-72686-0 PMC7512019 32968201

[26] Takamura M, Kurokawa K, Ootsuji H, Inoue O, Okada H, Nomura A, Kaneko S, Usui S (2017) Long-term administration of eicosapentaenoic acid improves post-myocardial infarction cardiac remodeling in mice by regulating macrophage polarization. J Am Heart Assoc 6:2 10.1161/JAHA.116.004560 PMC5523759 28223437

[27] Kalogeropoulos A, Georgiopoulou V, Psaty BM, Rodondi N, Smith AL, Harrison DG, Liu Y, Hoffmann U, Bauer DC, Newman AB (2010). Inflammatory markers and incident heart failure risk in older adults: the Health ABC (Health, Aging, and Body Composition) study. J Am Coll Cardiol.

[28] Bragazzi NL, Zhong W, Shu J, Abu Much A, Lotan D, Grupper A, Younis A, Dai H (2021) Burden of heart failure and underlying causes in 195 countries and territories from 1990 to 2017. Eur J Prev Cardiol 28(15):1682–1690 10.1093/eurjpc/zwaa147 33571994

[29] Tromp J, MacDonald MR, Tay WT, Teng TK, Hung CL, Narasimhan C, Shimizu W, Ling LH, Ng TP, Yap J, McMurray JJV, Zile MR, Richards AM, Anand IS, Lam CSP (2018) Heart failure with preserved ejection fraction in the young. Circulation 138(24):2763–2773 10.1161/CIRCULATIONAHA.118.034720 30565987

