# Supplementary material for: Does omega-3 supplementation improve the inflammatory profile of patients with heart failure? a systematic review and meta-analysis
Source: Heart Fail Rev. 2023 Jun 20;28(6):1417–25. doi: 10.1007/s10741-023-10327-0 (PMC10575807; doi:10.1007/s10741-023-10327-0)
Supplement: Supplementary file 1 — Supplementary file1 (DOCX 14 KB) [file 10741_2023_10327_MOESM1_ESM.docx]

**Table S1.**Search terms employed in the literature search.

| **Database** | **Search terms** |  |
| --- | --- | --- |
|  |  |  |
| PubMed | (Heart failure OR ejection fraction) AND (n-3 OR omega-3 OR EPA OR eicosapentaenoic acid OR DHA OR docosahexaenoic acid OR fish oil) AND (inflammat* OR TNF-a OR IL-* OR interleukin OR CRP OR VCAM-1 OR ICAM-1 OR MCP-1) |  |
| Cochrane Library | (Heart failure OR ejection fraction) AND (n-3 OR omega-3 OR EPA OR eicosapentaenoic acid OR DHA OR docosahexaenoic acid OR fish oil) AND (inflammat* OR TNF-a OR IL OR interleukin OR CRP OR VCAM-1 OR ICAM-1 OR MCP-1) |  |
| Scopus | (((heart AND failure) OR (ejection AND fraction) AND (omega-3 OR n-3) OR (eicosapentaenoic AND acid) OR (docosahexaenoic AND acid) OR (fish AND oil) inflammation AND (TNF-a OR interleukin OR CRP))) |  |
| Web of Science | (Heart failure OR ejection fraction) AND (n-3 OR omega-3 OR EPA OR eicosapentaenoic acid OR DHA OR docosahexaenoic acid OR fish oil) AND (inflammat* OR TNF-a OR IL-* OR interleukin OR CRP OR VCAM-1 OR ICAM-1 OR MCP-1) |  |
