# Supplementary figures and images for: Does omega-3 supplementation improve the inflammatory profile of patients with heart failure? a systematic review and meta-analysis
Source: Heart Fail Rev. 2023 Jun 20;28(6):1417–25. doi: 10.1007/s10741-023-10327-0 (PMC10575807; doi:10.1007/s10741-023-10327-0)

**Table S2.** Risk of bias assessment of the included clinical studies.


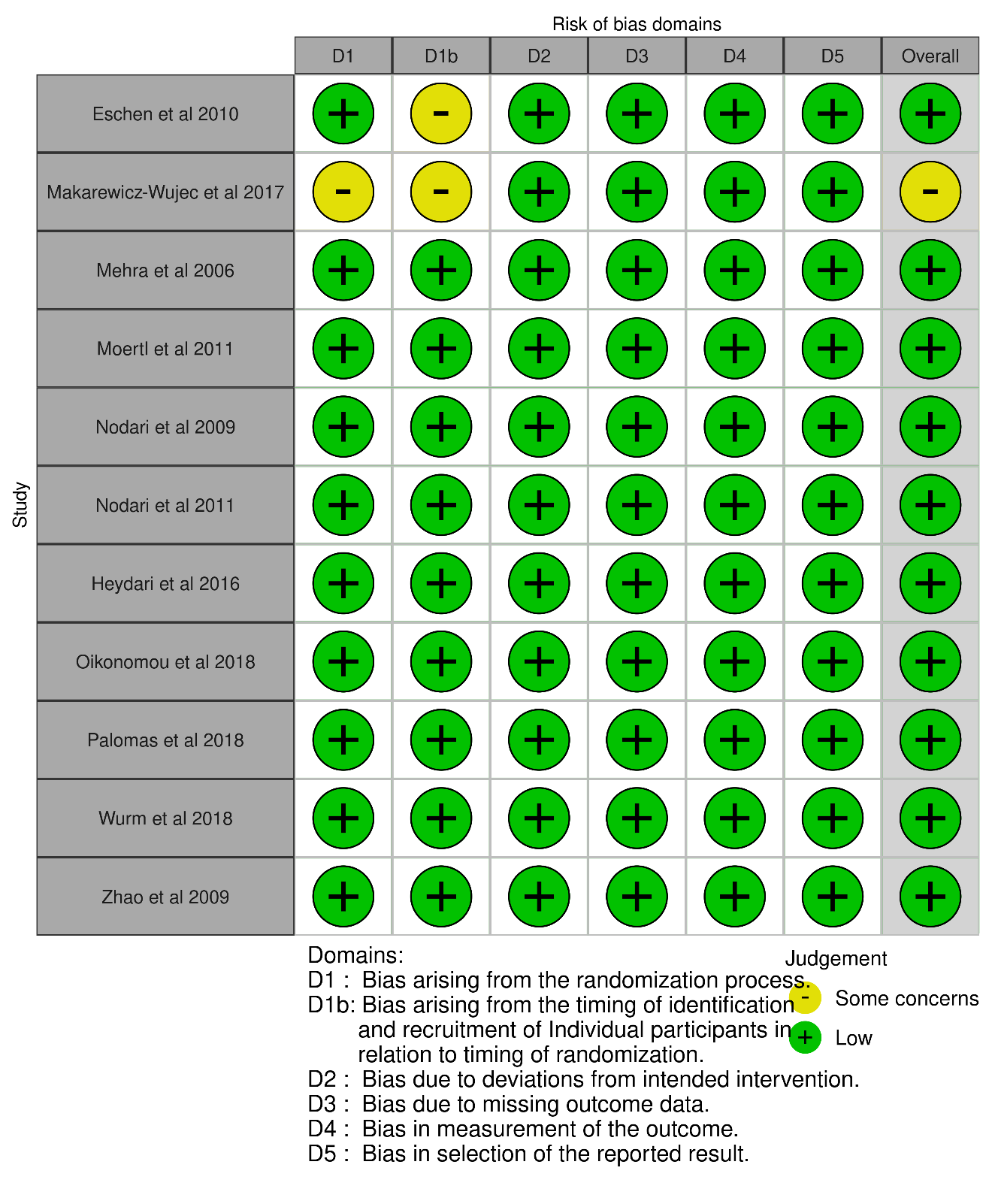

Supplement: Supplementary file 2 — Supplementary file2 (DOCX 566 KB) [file 10741_2023_10327_MOESM2_ESM.docx]

**Figure S10.** Effect of n-3 fatty acid supplementation on CRP levels in patients with LVEF < 40%.


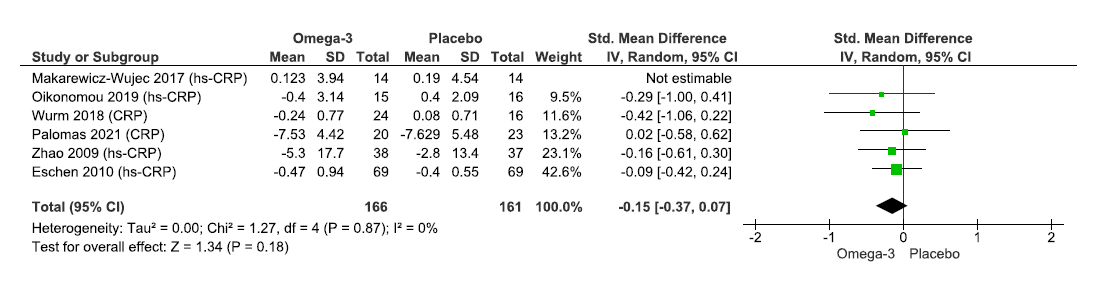

Supplement: Supplementary file 12 — Supplementary file12 (DOCX 55 KB) [file 10741_2023_10327_MOESM12_ESM.docx]

**Figure S12.** Effect of n-3 fatty acid supplementation on IL-1 levels in patients with HF.


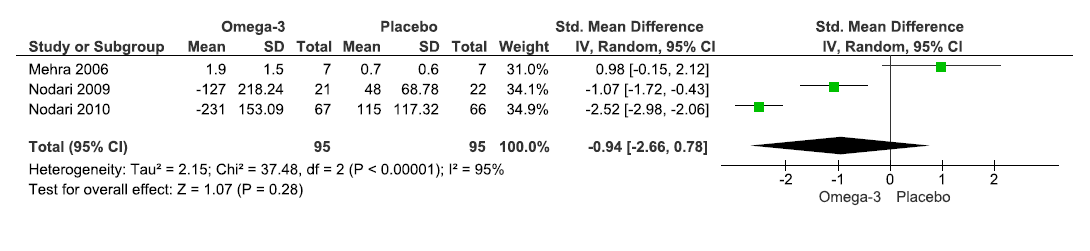

Supplement: Supplementary file 14 — Supplementary file14 (DOCX 43 KB) [file 10741_2023_10327_MOESM14_ESM.docx]

**Figure S13.** Effect of n-3 fatty acid supplementation on ICAM-1 and vICAM-1 levels in patients with HF.


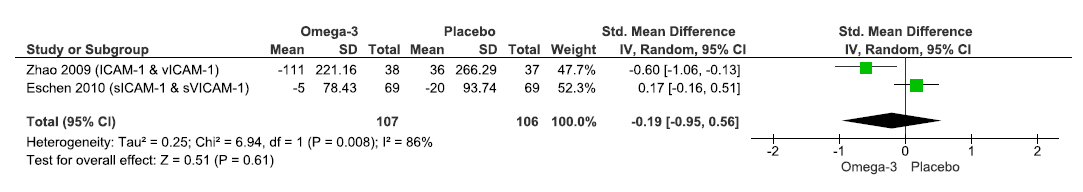

Supplement: Supplementary file 15 — Supplementary file15 (DOCX 38 KB) [file 10741_2023_10327_MOESM15_ESM.docx]
