# Supplementary material for: Does omega-3 supplementation improve the inflammatory profile of patients with heart failure? a systematic review and meta-analysis
Source: Heart Fail Rev. 2023 Jun 20;28(6):1417–25. doi: 10.1007/s10741-023-10327-0 (PMC10575807; doi:10.1007/s10741-023-10327-0)

**Figure S1.** Effect of n-3 fatty acid supplementation on TNF-a levels in patients with HF based on dose (<2 g. vs. ≥2 g.).


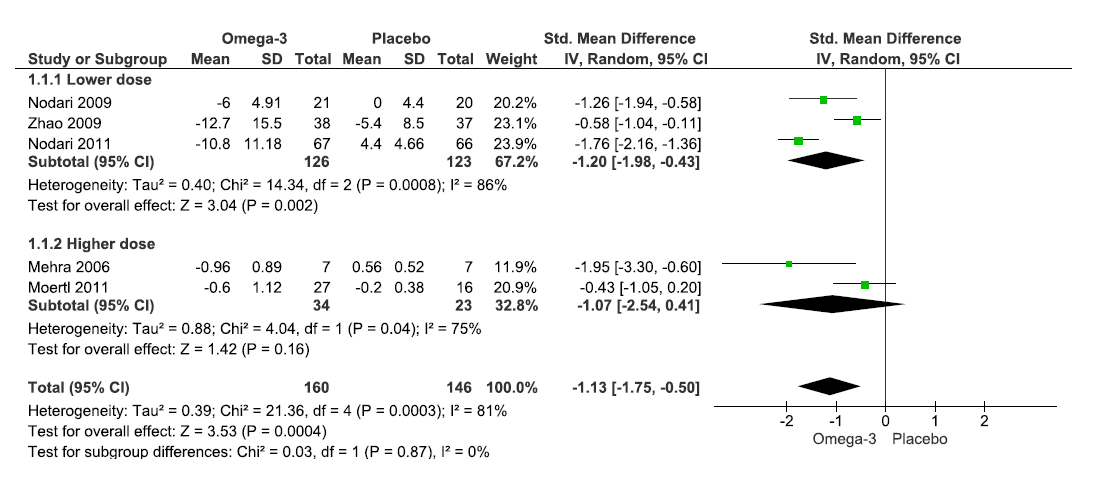

Supplement: Supplementary file 3 — Supplementary file3 (DOCX 75 KB) [file 10741_2023_10327_MOESM3_ESM.docx]
